# Effects of *Taiwanofungus camphoratus* on non-specific and specific immune activities in mice

**DOI:** 10.1080/21501203.2018.1437837

**Published:** 2018-02-21

**Authors:** Yun-Yu Chen, Chiu-Ping Lo, Chin-Chung Lin, Yi-Hsuan Hsieh

**Affiliations:** a Taiwan Leader Biotech Corp., Taipei City, Taiwan; b MedGaea Life Sciences Ltd., New Taipei City, Taiwan

**Keywords:** *Taiwanofungus camphoratus*, phagocytic activity, natural killer cells, specific immune response, non-specific immune response

## Abstract

*Taiwanofungus camphoratus* is a precious medicinal fungus endemic to Taiwan and has been used as traditional medicine for a long time. Many pharmacological studies have revealed that *T. camphoratus* possessed various biological activities, such as immunomodulatory effects, anticancer activity and liver protective function. The aim of this study is to investigate the non-specific and antigen (ovalbumin [OVA])-specific immunomodulation effects of solid-state cultivated powder of *T. camphoratus* (Leader *Antrodia cinnamomea* [LAC]) in BABL/c male mice. In non-specific and antigen-specific immune function studies, 8-week-old mice were orally administered with LAC for 6 and 8 weeks, respectively. The results have shown that the proliferation of splenic immune cells, phagocytic activity of macrophages and cytolytic activity of natural killer cells were enhanced by LAC. Additionally, LAC increased the levels of IL-2, TNF-α, INF-γ, GM-CSF and serum OVA-IgG and OVA-IgM. These findings provided evidences that LAC had the immunomodulation effects on both antigen-specific and non-specific immune responses in mice.

## 1. Introduction

*Taiwanofungus camphoratus* (syn. *Antrodia cinnamomea, Antrodia camphorata*) is an edible medicinal fungus native to Taiwan. It grows on the inner cavity of *Cinnamomum kanehirae* Hayata (Lauraceae) and has been used to promote health, treat liver disease, drug and food intoxication, hangover, exhaustion and cancers by the aboriginals in Taiwan for a long time (Wu SHR and Chang 1997; Geethangili and Tzeng 2011; Yue et al. 2012). Several bioactive compounds of *T. camphoratus* have been identified and the biological functions in immunomodulatory (Song et al. 2014; Kuo et al. 2008; Sheu et al. 2009), anticancer (Huang et al. 2015; Kumar et al. 2015; Lin et al. 2015), antiinflammation (Chen YF et al. 2014; Hsieh et al. 2010), antioxidant (Wu MD et al. 2011; Hseu et al. 2008) and hepatoprotective (Huang CH et al. 2010; Gokila Vani et al. 2014; Chang et al. 2016) effects were widely studied.

The regulation of immune system is important for human to fight with microorganism invasion and tumour cells, prevent autoimmune disease and ease allergy symptoms. Various studies have revealed mushrooms and mushroom polysaccharides possessed potent immunomodulatory effects and could induce different types of immune response. *Ganoderma lucidum* is able to trigger Th1 and Th2 cytokines expression and activate macrophages and T lymphocytes (Wasser 2002; Chan et al. 2005). *Agaricus bisporus* showed *in vivo* immunomodulatory effects on reducing the clinical signs of autoimmune encephalomyelitis in rats by regulation of the innate and adaptive responses (Ditamo et al. 2016). Oral administration of the extract of *Agaricus blazei* in mice increased the IgG level in serum, promoted phagocytic activity of peritoneal macrophages and natural killer (NK) cells activity (Ni et al. 2013). *T. camphoratus* also showed the effects on regulation of immune system and stimulating Th1 pathway in mice (Chen YJ et al. 2008; Cheng et al. 2008; Kuo et al. 2008). Besides, methyl antcinate K, the triterpenoids of *T. camphoratus* was demonstrated to activate dendritic cells and prime Th2 differentiation (Yu et al. 2009).

*T. camphoratus* has been widely used as food supplements and health food products in Taiwan. The purpose of this study is to evaluate the effects of solid-state cultivated powder of *T. camphoratus* (Leader *Antrodia cinnamomea* [LAC]) on non-specific and specific immune response and provide further insight of immunomodulatory effects of *T. camphoratus*. The results showed (198, 593, 988 mg/kg) oral administration of LAC in mice markedly increased the immune cell proliferation and enhanced phagocytic cell and NK cell activity. LAC also increased Th1 type cytokines secretion and the level of serum ovalbumin (OVA)-IgG and OVA-IgM.

## 2. Materials and method

### 2.1. LAC preparation

LAC capsule was manufactured by Taiwan Leader Biotech Corp. (Taipei, Taiwan), which contained 99% solid-state cultivated powder of *T. camphoratus* and 1% magnesium stearate. LAC was dissolved in sterile water to obtain dosing solutions of low (198 mg/kg bw), medium (593 mg/kg bw) and high (988 mg/kg bw) doses that were equivalent to one-, three- and fivefold recommended human daily intake, respectively.

### 2.2. Animals and treatments

BABL/c male mice were purchased from BioLASCO Taiwan Co., Ltd. (Taipei, Taiwan). Animals were housed at the laboratory animal centre of the MedGaea Life Sciences Institute under 12-h light/12-h dark cycle with free access to food and water. Animals were weighed once per week during the experimental period. For the non-specific immune tests, 8-week-old mice were divided into five groups of 10 mice each. Control group was treated with sterile water, positive control group was treated with the health food with immunomodulation effects on the market (260 mg/kg bw) and three treatment groups were treated with low, medium and high dose of LAC by oral gavage daily for 6 weeks. For the antigen-specific immune tests, animals were divided into five groups of 10 mice each and orally administration with LAC daily for 8 weeks. After 4 weeks of LAC treatment, each mouse was first immunised OVA by intraperitoneal injection of 50 μg/100 μl OVA (Sigma-Aldrich, St. Louis, MO, USA) emulsified in the Complete Freund’s Adjuvant (Sigma-Aldrich). Two weeks later, each mouse was intraperitoneally injected with OVA 100 μg/100 μl emulsified with Incomplete Freund’s Adjuvant (Sigma-Aldrich) to further to enhance the OVA-specific immune responses. Blood samples and spleen were collected at indicated time for further analysis.

### 2.3. Cell proliferation assay

The spleens were removed, and single-cell suspensions were cultured in RPMI 1640 medium. Splenocytes at a final density of 2.0 × 10^5^ cells/well were placed in 96-well plates. Cells were treated with 5 μg/mL concanavalin A (Con A), 10 μg/mL lipopolysaccharide (LPS) or 50 μg/mL OVA for 44 h. Cell proliferation assay was measured by using CellTiter 96® AQueous One Solution Cell Proliferation Assay Kit (Promega, Madison, WI, USA). In brief, after incubation, reagent containing a tetrazolium compound [3-(4,5-dimethylthiazol-2-yl)-5-(3-carboxymethoxyphenyl)-2-(4-sulfophenyl)-2H-tetrazolium, inner salt; MTS] and an electron coupling reagent (phenazine ethosulphate; PES) was added to each well. After 4 h incubation, 10% sodium dodecyl sulfate (SDS) was added to each well to stop the reaction, and the absorbance at 490 nm (OD_490_) was measured. The quantity of formazan product as measured by the amount of 490 nm absorbance is directly proportional to the number of living cells in culture. The stimulation index was calculated by the OD_490_ of mitogen-stimulated cells/OD_490_ of non-stimulated cells.

### 2.4. Cytokine analysis

The supernatants of Con A-, LPS- or OVA-stimulated cultured splenic cells were harvested after 24–48 h. The concentration of IL-2, IL-4, TNF-α, IFN-γ and GM-CSF cytokines in the culture supernatants was determined by ELISA (eBioscience, San Diego, CA, USA).

### 2.5. Phagocytic activity

To determine the phagocytes activity of mice treated with LAC, the granulocytes at a cell density of 1 × 10^6^ cells were cultured with FITC-labelled *E. coli* by using Phagocytosis analysis kit (Gene Research Lab. Co. Ltd., Taipei, Taiwan). Granulocytes were incubated with FITC-labelled *E. coli* in duplicate tubes (one at 4°C and the other at 37°C) for 15 min and analysed by flow cytometry. The proportion of phagocytosis (%) = *E. coli*^FITC^_37°C –_*E. coli*^FITC^_4°C_.

### 2.6. NK cell activity

Splenocytes (effector cells) were cultured with PHK67 green fluorescent labelled YAC-1 cells (target cells, 2 × 10^5^ cells) at effector: target ratios (E/T ratios) of 6.25:1 and 12.5:1, respectively. After 4 h incubation, cells were stained with propidium iodide and proportion of non-viable target cell (YAC-1 cell) was determined by flow cytometry.

### 2.7. Determination of serum OVA-IgG and OVA-IgM levels

Blood samples were collected from retro-orbital sinus of mice at the beginning of the study and in week 4, 5, 6, 7 and 8. After centrifuged at 3600 rpm for 10 min, the serum was collected for detection of anti-OVA-IgG and anti-OVA-IgM by ELISA. The level of anti-OVA-IgG and anti-OVA-IgM antibodies was calculated using the following formula:

ELISA unit (E.U.) = (OD_sample_ – OD_blank_)/(OD_positive serum_ – OD_blank_)

### 2.8. Statistical analysis

Results were presented as mean ± standard deviation. Statistical analysis was performed by one-way ANOVA followed by Duncan’s multiple range test using SPSS 13.0 software (SPSS Inc., Chicago IL). A value of *p *< 0.05 was considered as statistical significance.

## 3. Results

### 3.1. Proliferative activity of immune cells

In non-specific immune response tests, LAC at low, medium and high dose significantly increased the proliferation activity of splenocytes stimulated by Con A. However, no effect on LPS-stimulated immune cell proliferation (Table 1) was observed. In specific immune response tests, LAC had no effects on OVA-induced splenocytes proliferation.

**Table 1.** Effect of LAC on immune cell proliferation.

		Stimulation index		
Group	Dose (mg/kg)	Con A	LPS	OVA
NC	–	1.289 ± 0.121	1.521 ± 0.107	1.047 ± 0.057
LAC	198	1.486 ± 0.193*	1.572 ± 0.229	0.995 ± 0.075
LAC	593	1.595 ± 0.149*	1.622 ± 0.135	1.002 ± 0.069
LAC	988	1.438 ± 0.129*	1.568 ± 0.208	1.007 ± 0.040
PC =	260	1.450 ± 0.157*	1.523 ± 0.198	1.037 ± 0.056

All data were presented as mean ± SD. NC: normal control; PC: positive control. LAC: solid-state cultivated powder of *T. camphoratus*. *p < 0.05 compared to normal control.

### 3.2. Effect of LAC on NK cell activity

**Figure 1.**
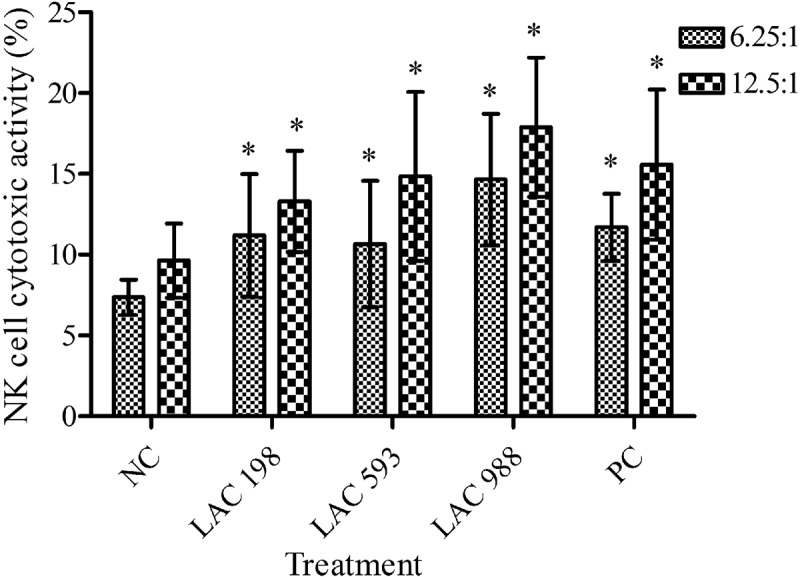
Effect of LAC on natural killer (NK) cell activity. The mice were administered with LAC (198, 593, 988 mg/kg) and positive control (PC, 260 mg/kg) for 6 weeks. The spleen cells were isolated from mice and NK cell activity was analysed by cytotoxic assay. The effector-to-target ratios (E/T ratios) were 6.25:1 and 12.5:1. All data were presented as mean ± SD. *p < 0.05 compared to normal control (NC).

LAC at low, medium and high dose obviously increased the proportion of YAC-1 cell death at both E/T ratios of 6.25:1 and 12.5:1 in a dose-dependent manner (Figure 1). The results suggested that LAC effectively enhanced NK cell activity.

### 3.3. Effect of LAC on phagocytes activity

**Figure 2.**
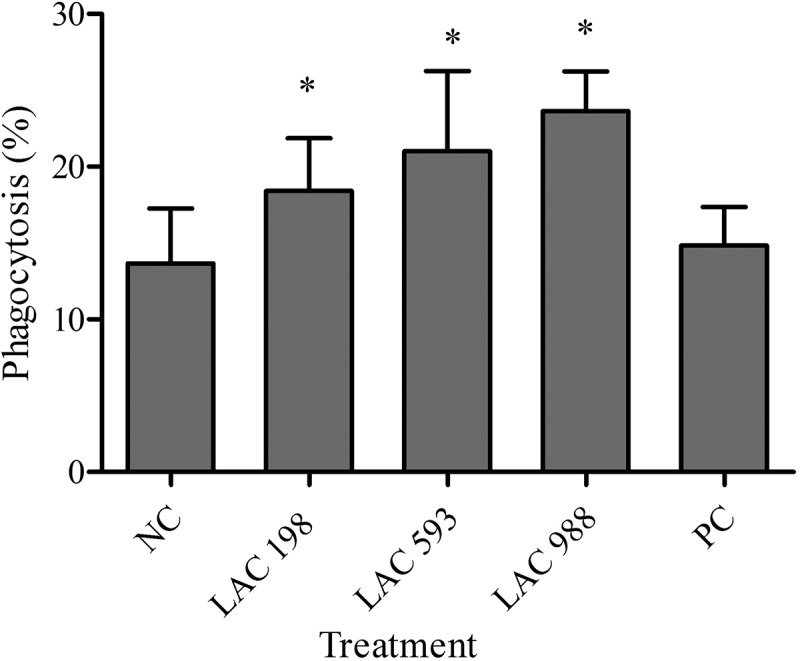
Effect of LAC on phagocytic cell activity. The mice were administered with LAC (198, 593, 988 mg/kg) and positive control (PC, 260 mg/kg) for 6 weeks. The spleen cells were isolated from mice and phagocytosis of *E. coli* by leukocytes was analysed by flow cytometry. All data were presented as mean ± SD. *p < 0.05 compared to normal control (NC).

The percent of phagocytosis of LAC groups at low (18.42 ± 3.45), medium (22.23 ± 5.84) and high dose (23.61 ± 2.71) was significantly increased compared to normal control (13.67 ± 3.59) and with the dose-dependent effect. The results of positive control (14.49 ± 2.45) showed no difference versus normal control (Figure 2).

### 3.4. Effect of LAC on cytokines level

In non-specific immune study model, the splenocytes were isolated and stimulated by Con A and LPS. The level of IL-2, IL-4, TNF-α, IFN-γ and GM-CSF was analysed. As shown in Table 2, there was no significance change without mitogen stimulation among all groups. The level of IL-2, TNF-α, IFN-γ and GM-CSF in all LAC-treated groups showed markedly increased while stimulated by Con A and LPS. In specific immune study model, the splenocytes were isolated and stimulated by Con A and OVA and the results showed LAC markedly increased the level of IL-2, TNF-α, IFN-γ and GM-CSF in splenocytes stimulated by Con A. LAC only significantly increased IL-2 and IFN-γ in splenocytes stimulated by OVA. IL-4 showed no significance change among all groups (Table 3).

**Table 2.** Effect of LAC on cytokines secretion in non-specific immune response.

Group	Dose (mg/kg)	Con A (5 μg/mL)	LPS (10 μg/mL)
		IL-2 (pg/mL)	
NC	–	221.44 ± 56.47	53.37 ± 9.86
LAC	198	293.43 ± 67.66*	86.92 ± 17.35*
LAC	593	291.99 ± 61.01*	85.73 ± 15.81*
LAC	988	307.87 ± 45.74*	86.35 ± 23.85*
PC	260	262.87 ± 51.48	69.70 ± 21.97
		IL-4 (pg/mL)	
NC	–	21.25 ± 9.80	4.92 ± 1.34
LAC	198	21.28 ± 7.19	7.28 ± 3.36
LAC	593	19.10 ± 5.90	7.25 ± 3.70
LAC	988	17.72 ± 4.30	7.53 ± 2.89
PC	260	14.86 ± 7.50	5.09 ± 1.80
		TNF-α (pg/mL)	
NC	–	313.93 ± 107.64	221.79 ± 90.35
LAC	198	410.36 ± 82.17*	331.79 ± 125.30
LAC	593	410.24 ± 106.57*	433.81 ± 176.07*
LAC	988	417.19 ± 77.95*	410.00 ± 138.37*
PC	260	442.19 ± 90.03*	293.08 ± 77.07
		IFN-γ (pg/mL)	
NC	–	1329.82 ± 345.51	186.23 ± 58.04
LAC	198	1698.73 ± 323.08*	204.77 ± 67.81
LAC	593	1690.90 ± 360.44*	276.86 ± 97.37*
LAC	988	1765.42 ± 207.41*	270.92 ± 109.60*
PC	260	1737.08 ± 210.89*	199.36 ± 76.81
		GM-CSF (pg/mL)	
NC	–	226.52 ± 62.19	21.04 ± 4.13
LAC	198	290.70 ± 42.21*	25.33 ± 6.32
LAC	593	317.65 ± 95.27*	31.47 ± 7.95*
LAC	988	316.36 ± 68.19*	29.57 ± 9.55*
PC	260	214.17 ± 70.38	21.96 ± 7.85

All data were presented as mean ± SD. NC: normal control; PC: positive control. LAC: solid-state cultivated powder of *T. camphoratus*. *p < 0.05 compared to normal control.

**Table 3.** Effect of LAC on cytokines secretion in specific immune response.

Group	Dose (mg/kg)	Con A (5 μg/ml)	OVA (50μg/ml)
		IL-2 (pg/ml)	
NC	–	125.4 ± 41.6	9.5 ± 3.8
LAC	198	241.3 ± 81.5*	14.4 ± 3.9*
LAC	593	234.9 ± 85.8*	15.1 ± 3.7*
LAC	988	239.7 ± 66.3*	15.7 ± 5.5*
PC	260	144.9 ± 57.4	10.2 ± 4.2
		IL-4 (pg/ml)	
NC	–	17.1 ± 4.5	11.5 ± 3.1
LAC	198	18.9 ± 7.2	12.0 ± 2.5
LAC	593	16.0 ± 6.9	11.2 ± 4.8
LAC	988	16.1 ± 5.9	11.0 ± 3.9
PC	260	14.4 ± 5.1	11.0 ± 4.5
		TNF-α (pg/ml)	
NC	–	145.5 ± 27.7	26.3 ± 8.2
LAC	198	462.5 ± 151.8*	37.5 ± 4.3
LAC	593	449.2 ± 163.4*	35.0 ± 20.4
LAC	988	456.8 ± 183.8*	31.9 ± 12.9
PC	260	196.7 ± 75.1	20.7 ± 7.9
		IFN-γ (pg/ml)	
NC	–	688.2 ± 140.3	22.1 ± 7.0
LAC	198	1124.4 ± 529.8*	34.0 ± 7.4*
LAC	593	1118.2 ± 534.2*	36.2 ± 7.9*
LAC	988	1287.4 ± 583.6*	33.3 ± 7.8*
PC	260	709.2 ± 219.2	20.9 ± 4.8
		GM-CSF (pg/ml)	
NC	–	52.8 ± 13.3	3.2 ± 3.5
LAC	198	81.9 ± 15.5*	2.3 ± 2.3
LAC	593	81.1 ± 17.8*	3.2 ± 3.0
LAC	988	81.5 ± 20.7*	3.5 ± 2.6
PC	260	60.3 ± 13.2	4.4 ± 2.6

All data were presented as mean ± SD. NC: normal control; PC: positive control. LAC: solid-state cultivated powder of *T. camphoratus*. *p < 0.05 compared to normal control.

### 3.5. Effect of LAC on serum antibody secretion

**Figure 3.**
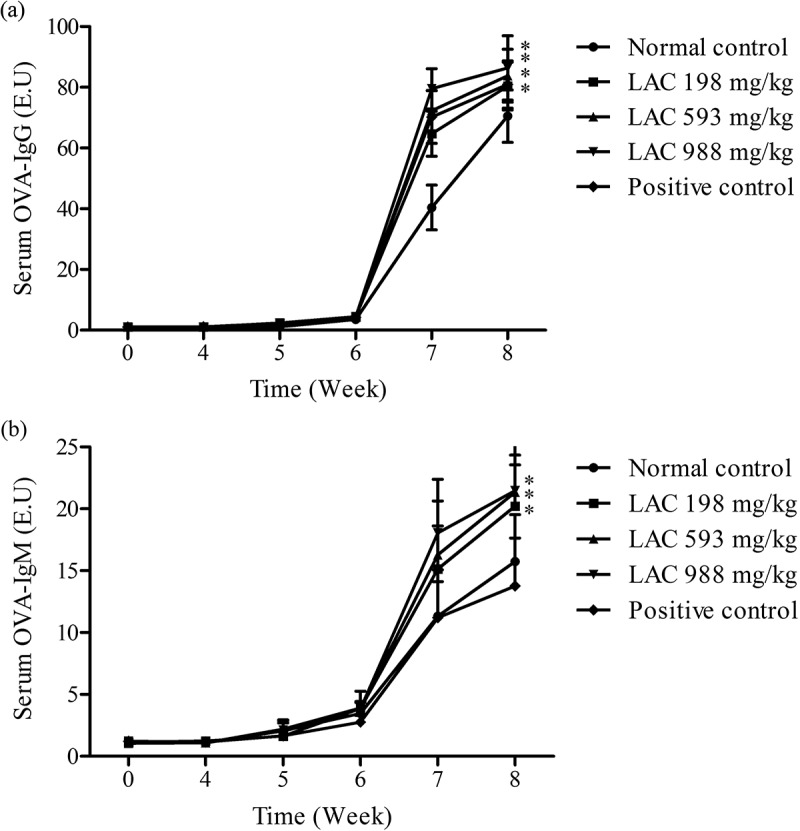
Effect of LAC on serum OVA-IgG and OVA-IgM secretion. The mice were administered with LAC (198, 593, 988 mg/kg) and positive control (PC, 260 mg/kg) for 8 weeks. (a) The serum OVA-IgG and (b) OVA-IgM were analysed using ELISA assays. All data were presented as mean ± SD. *p < 0.05 compared to normal control (NC).

The serum OVA-IgG and OVA-IgM were determined after mice were immunised by OVA in week 4. The results showed both OVA-IgG and OVA-IgM were increased as time increased in each group after OVA stimulation compared to non-stimulation. There was a markedly increased after the second OVA immunisation (week 6). LAC significantly increased the level of serum OVA-IgG and OVA-IgM in week 4–8 and week 7–8, respectively and with the dose-dependent effect (Figure 3).

## 4. Discussion

*T. camphoratus* has demonstrated the immunomodulatory effects in various studies. Most of these findings indicated polysaccharides from *T. camphoratus* mycelium had the great potential to develop as immunomodulatory agent to treat infection disease or as a vaccine adjuvant for cancer therapy (Chen et al. 2008; Cheng et al. 2008; Kuo et al. 2008; Lin et al. 2015). In this study, we found LAC, composed of solid-state cultivated powder of *T. camphoratus*, had the ability to regulate non-specific and specific immune response. There was no effect on body weight, organ weight and no obvious clinical signs observed in mice administered with LAC (data not shown). These results suggested orally gavage of LAC at dose up to 988 mg/kg bw showed no toxic in mice.

For non-specific immune study, mice fed with LAC showed increasing immune cell proliferation under stimulation by Con A. Con A was a stimulator that selectively induced T cell proliferation and LPS is a potent mitogen of B cells. The results suggested that LAC had the ability on enhancing T cell proliferation but no effect on stimulating B cell proliferation. Besides, LAC enhanced NK cell activity and phagocytic cell activity in a dose-dependent manner. NK cell and phagocytes play important roles in innate immunity. Kuo et al. have indicated that *T. camphoratus* could increase phagocytic activity of leucocytes, monocytes and human polymorphonuclear neutrophils. Polysaccharides and adenosine might be the main components of *T. camphoratus* that contribute to immunomodulatory effects (Kuo et al. 2008). Our finding was consistent with this study. LAC was the solid-state cultivated powder of *T. camphoratus,* which contains many bioactive components such as polysaccharides and adenosine. This suggested LAC had the potential to increase innate immune responses to protect body from microbial infection. However, which components were the major active factors need to be clarified in further studies.

Various researches have indicated polysaccharides isolated from *T. camphoratus*-induced Th1 type cytokines, IL-2, TNF-α and IFN-γ, and promote T cell towards Th1 pathway. Besides, *T. camphoratus* has been found to prevent asthma and inhibit *Schistosoma mansoni* infection by enhancing Th1 responses (Wasser 2002; Chen et al. 2008; Cheng et al. 2008; Lu et al. 2009). In this study, it was found that LAC increased IL-2, TNF-α, IFN-γ and GM-CSF in spleen cells under stimulation by Con A and LPS. Moreover, the function of these cytokines could promote T cell proliferation and activate macrophages and NK cell (Gaffen and Liu 2004; Broere et al. 2011). These findings suggested that LAC increased T cell proliferation and NK cell activity through inducing Th1 type cytokines and appeared to trigger immune system developing to Th1 response. The present study also found LAC regulated specific immune response. The results showed LAC increased serum IgG, IgM levels in mice after OVA immunisation. Additionally, LAC increased OVA-stimulated IL-2 and IFN-γ secretion, but had no effect on immune cell proliferation after OVA immunisation. According to these observations, LAC showed the ability to modulate cellular immunity and humoral immunity.

In conclusion, LAC exhibited the effects on increasing immune cell proliferation, enhancing phagocytes and NK cell activity. However, the exact mechanism needs to be explored in the future studies. This study provided evidences that LAC, solid-state cultivated powder of *T. camphoratus*, had immunomodulatory effects and is safe for further human use.
